# Range Spectral Filtering in SAR Interferometry: Methods and Limitations

**DOI:** 10.3390/s22228696

**Published:** 2022-11-10

**Authors:** Alejandro Mestre-Quereda, Juan M. Lopez-Sanchez, Jordi J. Mallorqui

**Affiliations:** 1 Hisdesat Strategic Services S.A., 28046 Madrid, Spain; 2 Signals, Systems and Telecommunications Group, Institute for Computer Research (IUII), University of Alicante, 03080 Alicante, Spain; 3 CommSensLab, Universitat Politecnica de Catalunya, 08034 Barcelona, Spain

**Keywords:** SAR interferometry, range filtering, spectral shift

## Abstract

A geometrical decorrelation constitutes one of the sources of noise present in Synthetic Aperture Radar (SAR) interferograms. It comes from the different incidence angles of the two images used to form the interferograms, which cause a spectral (frequency) shift between them. A geometrical decorrelation must be compensated by a specific filtering technique known as *range filtering*, the goal of which is to estimate this spectral displacement and retain only the common parts of the images’ spectra, reducing the noise and improving the quality of the interferograms. Multiple range filters have been proposed in the literature. The most widely used methods are an *adaptive* filter approach, which estimates the spectral shift directly from the data; a method based on *orbital* information, which assumes a constant-slope (or flat) terrain; and *slope-adaptive* algorithms, which consider both orbital information and auxiliary topographic data. Their advantages and limitations are analyzed in this manuscript and, additionally, a new, more refined approach is proposed. Its goal is to enhance the filtering process by automatically adapting the filter to all types of surface variations using a multi-scale strategy. A pair of RADARSAT-2 images that mapped the mountainous area around the Etna volcano (Italy) are used for the study. The results show that filtering accuracy is improved with the new method including the steepest areas and vegetation-covered regions in which the performance of the original methods is limited.

## 1. Introduction

Synthetic Aperture Radar (SAR) interferometry (InSAR) has been established as a powerful remote sensing technique for mapping the Earth’s surface. InSAR applications include the generation of highly accurate topographic models, estimation of land subsidence, and analysis and monitoring of seismic deformations or volcanic activities [1].

Interferometric methods exploit the phase information of interferograms, which are formed by combining two coregistered SAR images from the same area. Unfortunately, SAR interferograms are affected by different decorrelation sources (i.e., noise) that degrade the quality of the final products [2]. They are briefly introduced here for the sake of completeness. A *miss-registration decorrelation* is caused by errors in the coregistration of the two SAR images. It can be avoided by an accurate registration at the sub-pixel level, so it is usually negligible. An *SNR (signal-to-noise ratio) decorrelation* is induced by the thermal noise present in the electronic systems located in the SAR instrument. It is noticeable in areas with low backscatter for which the SNR is low. A *volume decorrelation* comes from the presence of scattering elements at different heights inside the SAR resolution cell, such as in the presence of a vegetation volume (e.g., in forests). A *temporal decorrelation* is due to changes in the physical and geometrical properties of the imaged areas, which are produced from the acquisition of the two SAR images. Note that both volume and temporal decorrelations cannot be specifically compensated. A *Doppler decorrelation* is due to variations in the antenna direction between the two acquisitions, which cause a different frequency shift in the azimuth spectrum (Doppler Centroid). This source of decorrelation can be appropriately compensated by using a process known as azimuth filtering, the goal of which is to remove the non-overlapping frequency bands in the azimuth dimension [1,3]. The last source of decorrelation, which constitutes the focus of this work, is the *geometrical (or baseline) decorrelation*. It is caused by the InSAR acquisition geometry, that is, two SAR acquisitions obtained from slightly different positions. This difference in the incidence angles causes a frequency shift between the range spectra of the two images. When forming the interferogram, only the common band contributes to the interferometric phase, whereas the rest only contribute noise. This frequency shift depends not only on the satellite look angle difference but also on the local terrain slope [4].

This has led to the conception of range-filtering strategies in frequency domains, which first, have to accurately estimate the spectral shift and second, properly remove the non-common band of the spectra while preserving the useful (common) part. The filtering step implies a degradation of the spatial resolution along the range. Without this range filtering, the coherence, and hence the global quality of the interferogram, would decrease proportionally to the perpendicular baseline (or equivalently to the local incidence angle difference). The suppression of a baseline decorrelation is, therefore, important for improving the quality of products derived directly from the interferometric phase (e.g., surface deformation). Moreover, there are techniques that exploit the coherence magnitude as an input feature for the retrieval of biophysical variables. A typical example is the estimation of vegetation height based on considering the input coherence as a direct measure of the volume decorrelation [5,6,7,8]. These techniques assume that the input coherence depends only on the volume decorrelation and is free from any other decorrelation sources (including baseline decorrelation). Therefore, the objective of range filtering is restricted not only to increase the coherence for obtaining a better phase quality but also to estimate the coherence correctly (i.e., free from other decorrelation sources).

This paper is organized as follows. First, Section 2.1 analyzes the key aspects of range filtering for InSAR. The frequency-domain explanation of interferometry is briefly introduced to justify the need for range filtering. Then, the different range filters conceived in the past are explained in Section 2.2, Section 2.3.1 and Section 2.3.2, whereas Section 2.3.3 presents the proposed method. The good performance of the new method compared with those of the classical methods is evaluated in Section 3, highlighting both the advantages and limitations of each one.

## 2. Theoretical Background and Methods

### 2.1. Generalities

Range filtering is usually carried out right after images’ coregistration [9,10] and is regarded as a pre-processing step because it is applied before interferogram formation. The filtering addresses the inherent loss of coherence induced by the difference between the incidence angles of master and slave images that form the interferogram [4,11]. Larger baselines result in higher decorrelation levels to the extent that there is a limit, known as a *critical baseline* [3], that leads to fully decorrelated interferograms. Its expression can be geometrically derived from the system parameters as

(1) $$B_{⊥,crit}=\frac{B_{W}\lambda \tan\left(\theta -\alpha \right)}{c},$$

where $B_{W}$ is the range bandwidth, λ is the sensor wavelength, θ is the incidence angle, α is the local terrain slope, *c* is the speed of light, and *R* is the distance from the satellite position to the ground.

**Figure 1:**
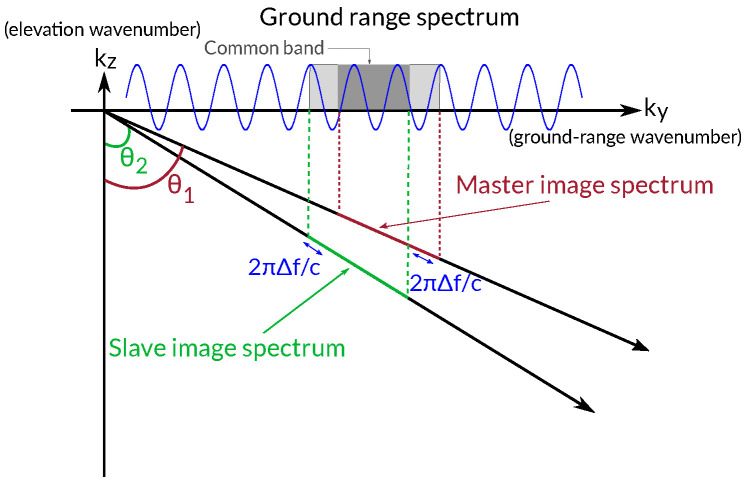
Illustration of the wavenumber shift principle.

The most rigorous explanation of spatial decorrelation can be conducted in the spectral domain. As a consequence of the slightly different positions of the sensor when the two images were acquired, each image samples a different part of the ground reflectivity spectrum. The difference is known as the *wavenumber shift*, as represented in Figure 1.

For filtering purposes, it is worth expressing this wavenumber shift as an equivalent frequency shift Δf as a function of the acquisition parameters [4].

(2) $$\Delta f=\frac{f_{0}\Delta \theta }{\tan(\theta_{1}-\alpha )},$$

where $f_{0}$ is the carrier frequency, $\theta_{1}$ and $\theta_{2}$ are the look angles of the reference (master) image and the slave image, respectively, $\Delta \theta =\theta_{1}-\theta_{2}$ is the local incidence angle difference, and α is the local terrain slope. Moreover, the angular separation Δθ can be approximated by

(3) $$\Delta \theta \approx \frac{B_{⊥}}{R},$$

where $B_{⊥}$ is the perpendicular baseline and *R* the range, so the frequency shift can be expressed as

(4) $$\Delta f\approx \frac{f_{0}B_{⊥}}{R\tan(\theta_{1}-\alpha )}=\frac{cB_{⊥}}{\lambda R\tan(\theta_{1}-\alpha )}.$$

**Figure 2:**
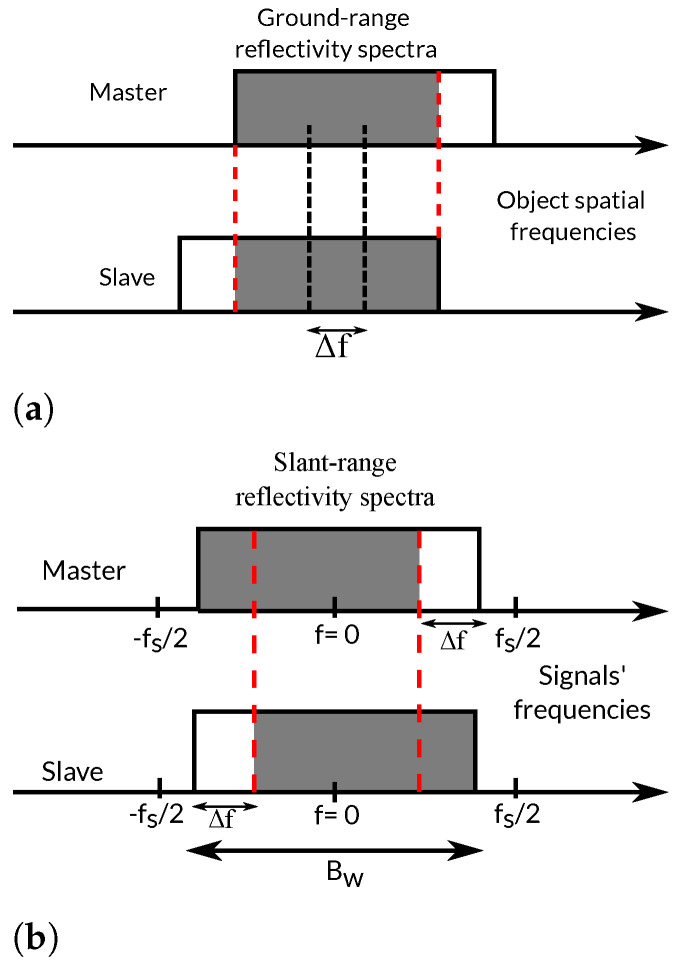
Illustration of the spectral shift (ground-range (**a**) and slant-range domains (**b**)) between two images. Only the common band (filled in gray) contains useful interferometric information.

The amount of spectral shift provides an indication of the degree of decorrelation between both images. In this regard, the perpendicular baseline that produces a frequency shift Δf equal to the range bandwidth is the *critical baseline*. Any interferogram obtained from image pairs with baselines larger than the critical baseline will be fully decorrelated since the images’ spectra would be completely disjoint (see Figure 1). For smaller baselines, and as illustrated in Figure 2, only the common band contributes constructively to the interferogram, whereas the non-common band just contributes noise. Note that the measured slant-range spectrum only depends on the transmitted bandwidth and sampling frequency (and hence is common for both images), but the ground-range spectra of the images are mapped on different slant-range spectral positions. It is clear that a proper filtering that cancels out the non-common bands can improve the interferogram quality but at the price of a reduction in the range resolution.

This has led to the conception of range-filtering strategies in frequency domains, which have to accurately remove the non-common spectral bands while preserving as much as possible the useful (common) part. It is important to note that the spectral shift is not uniform along the scene and that it strongly depends on the local topography.

Different spectral filtering methods have been conceived in the past. The goal of all of them is an appropriate estimation of the frequency shift along the scene. Once the shift is estimated, a band-pass filter is built in order to remove the non-overlapping parts of the spectra. The main difference between the methods is how the shift is estimated. The rest of the filtering steps are common and can be summarized as follows:

1. The spectral weighting applied to each image (during the image focusing) in order to limit the side-lobe contributions of the strong point targets is removed to retrieve the original flat range spectrum  [12,13,14].
2. The spectral shift Δf is estimated according to each method and, once obtained, a modified spectral weighting window is defined that eliminates the non-common parts of the spectra.
3. This leads to a new and reduced range bandwidth $B_{W^{\prime }}$, which is a function of Δf
  (5) $$B_{W^{\prime }}=B_{W}-\left|\Delta f\right|,$$
  with a subsequent degradation of the spatial resolution in the range, but with an increase in the correlation between both images.

In the following subsections, the main filtering algorithms proposed in the past are explained.

### 2.2. Adaptive Method

The *adaptive* method is widely used [1,15,16]. It has the advantage of not requiring any external information since the spectral shift between both images is directly estimated from them. However, a sufficient initial degree of correlation (coherence) is required between the images since a preliminary interferogram is used to compute the local spectral displacement. As a consequence, this method provides good results in already coherent areas but performs poorly if other sources of decorrelation are present, especially when a temporal decorrelation worsens the quality of the data. This method also shows poor performance in vegetated areas, even in single-pass acquisitions when a volume decorrelation drives the coherence.

The core idea of this method relies on computing the power spectrum (range dimension) of the complex interferogram and performing a peak analysis. Prior to interferogram formation, both the master and slave images should be oversampled by a certain factor to increase the precision of the peak location (usually, a factor of 2 or 4 is enough). The spectral shift is directly provided by the location of the peak. In practice, the algorithm works in blocks, i.e., the image is divided into small overlapped patches with a given number of pixels and lines. Although each line of the block is individually evaluated, a fixed number of lines inside the block is averaged in order to reduce the interferogram noise. For instance, if the block has 500 lines, 25 lines can be averaged to filter the central one. Once the peak is located by the maximum value of the power spectrum, a pseudo-SNR is also calculated. This signal-to-noise ratio is directly related to the ’quality’ of the peak, i.e., whether it is clearly identified and can be considered a reliable measure of the spectral displacement or, contrarily, it is just a local maximum value extracted from a low-quality (decorrelated) area, hence not a reliable measure. The SNR can be computed as

(6) $$SNR=\frac{N\cdot |X_{p_{max}}|}{\sum_{k\neq p_{max}}\left|X_{k}\right|},$$

where *N* is the number of range samples in the block, *k* is the sample position, $X_{p_{max}}$ is the maximum value of the power spectrum, and |X| is the averaged spectrum in the range dimension of the interferogram. A threshold is therefore set to determine if the estimated spectral shift is reliable or not. Only if the SNR is above the threshold will the range be filtered. Otherwise, the range line is not modified.

At this point, it is worth mentioning the influence of topography variations (represented by the variations in the local terrain slopes) on the filtering process. Since the peak corresponds to the maximum value of the Fourier transform of the interferogram, it can be regarded as the dominant frequency inside the range block. In other words, the local frequency shift is equal to the fringe frequency. Accordingly, if the slopes change significantly, the peak will inevitably widen and will not be representative of a single frequency shift.

Consequently, the filter would ideally require the slope to be perfectly constant inside the extracted block. In this regard, the filter can be tuned so that small interferogram patches (with fewer pixels in the range dimension) are processed when the topography presents strong variations. In this case, even though the filter would be better adapted to the local topography, the reduced number of samples would lead to a worse spectral resolution and a coarser estimation of the spectral shift. However, for simplicity and to reduce the computational cost of the method, the use of adaptive filtering window sizes is not considered and a constant block size of 64 or 128 pixels (in a range) is employed.

### 2.3. DEM-Based Filtering

The rest of the methods make use of orbital information and a digital elevation model (DEM) of the imaged area to compute the frequency shift on the basis of the observation geometry, but there are different approaches, which are detailed in the following subsections.

#### 2.3.1. Method Based on a Constant DEM

The first method based on orbital information assumes a flat-Earth model so the local terrain slope is kept constant at zero. Then, it employs the local perpendicular baseline derived from the orbit state vectors to compute the spectral displacement using Equation (4), assuming α=0. The filtering is usually performed line by line (i.e., by extracting the whole range for every azimuth position), where each line is, in turn, divided into smaller blocks. Blocks of 128 or 256 pixels are recommended. Then, a single spectral shift within each block of the interferogram is computed. In order to improve filtering performance, the largest spectral shift is selected among all the obtained values (128 or 256 depending on the block size).

The zero-slope assumption leads to sub-optimal filtering performance in areas with a steep topography since the local slope variations in the surface are not considered. Therefore, ignoring topography constitutes the main limitation of this method.

#### 2.3.2. Slope-Adaptive Filtering with External DEM

Since local topography plays an important role in determining the spectral shift, the next method is based on the exploitation of an auxiliary DEM, which contains the terrain height from which the local slopes can be estimated. We call this method *slope-adaptive* filtering. In principle, it is based on the approach discussed in Section 2.3.1 but takes into account the slopes derived from an auxiliary DEM, i.e., α is different from zero.

The inclusion of elevation information was initially proposed in [17] (see also the companion patent [18]). Since the spectral shift is equal to the fringe frequency caused by the topography in the space domain, the estimated frequencies can be used to filter in the range. Although the proposed methodology has the advantage of being completely automatic (everything is derived from the pair of images), it entails the risk of not being accurate enough in areas with low-quality and decorrelated data, where the estimation of frequencies is a very difficult task. Nevertheless, the slope-adaptive filtering scheme proposed in [17] can be easily adapted to use an external DEM instead of estimating it from the original data. In fact, an external DEM is usually considered in InSAR processing for the coregistration [9], that is, a DEM of the imaged area is commonly available, such as the global SRTM DEM [19]. The only step necessary here is the reprojection of the DEM (originally in cartographic coordinates) to the SAR coordinates (slant-range/azimuth) using the so-called inverse geocoding or back-geocoding [1] method. As a result of the process, we obtained the height of every pixel of a SAR image.

A simple procedure for including a DEM in the filtering process relies on performing a demodulation of the master and slave images. This can be directly achieved using the synthetic interferogram derived from the DEM and orbits, which contains the topographic phase contribution. Thus, both of the original SAR images $S_{1}$ and $S_{2}$ are demodulated according to

(7) $$\begin{array}{c} \begin{array}{cc} S{{}_{1}}^{\prime } & =S_{1}\exp\;\left(-j\;\frac{\phi_{DEM}}{2}\right),\\ S{{}_{2}}^{\prime } & =S_{2}\exp\;\left(+j\;\frac{\phi_{DEM}}{2}\right), \end{array} \end{array}$$

where $\phi_{DEM}$ is the topographic phase derived from the DEM. Note that half of the topographic phase contribution is used for the master image and the other half for the slave image but with opposite signs.

The next step consists of computing the spectral displacement, which can be directly estimated using Equation (4). As the demodulation of the images has aligned their spectra, a low-pass filter with the proper bandwidth has to be applied to eliminate the non-common parts. The bandwidth of the filter depends, among other factors, on the local slope and thus changes along the scene. The simplest procedure consists of filtering the whole images or blocks by a constant Δf, usually the maximum shift. The images can be low-pass filtered employing the same symmetric filter in the frequency domain. This approach does not fully exploit the DEM information as it over-filter areas with mild topography. The strategy can be improved by locally applying the proper filtering on the overlapped patches. More details regarding the implementation are provided in the next section.

Finally, the topographic phase removed in Equation (7) must be added back to the images, leading to the range-filtered images $S_{1f}$ and $S_{2f}$ expressed as

(8) $$\begin{array}{c} \begin{array}{cc} S_{1f} & =S_{1}^{\prime }\exp\;\left(+j\;\frac{\phi_{DEM}}{2}\right),\\ S_{2f} & =S_{2}^{\prime }\exp\;\left(-j\;\frac{\phi_{DEM}}{2}\right). \end{array} \end{array}$$

**Figure 3:**
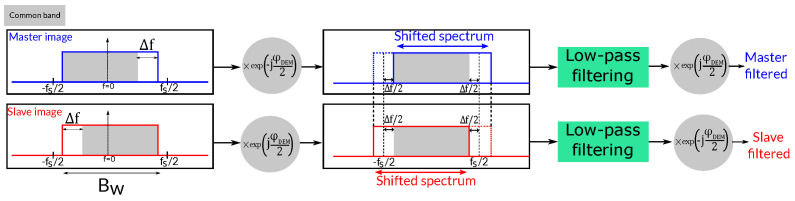
Common band alignment of two images’ spectra using demodulation with a topographic phase, followed bylow-pass filtering.

This process is shown in Figure 3.

#### 2.3.3. Multi-Scale Slope-Adaptive Filtering with External DEM

Once the different state-of-the-art range-filtering strategies have been reviewed, a refined method is proposed in this section. The core idea consists of providing different options to overcome the limitations of the previously explained filters using the complete exploitation of an external DEM. It is important to state that we focus on the problems that commonly-used filters show in areas with steep and/or varying topography or with a strong temporal and/or volume decorrelation. Otherwise, the previous methods provide acceptable results, as the spectral shift estimation is not influenced by these factors.

**Figure 4:**
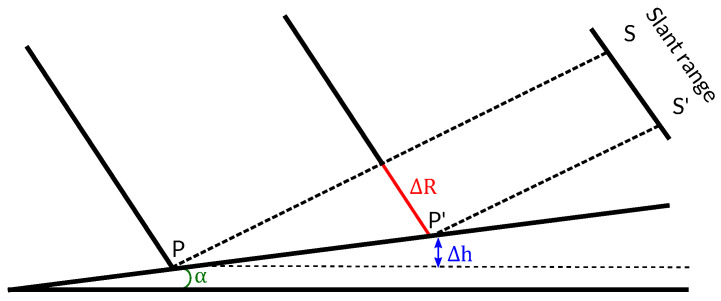
Local terrain slope (α) acquisition geometry between two adjacent points (*S* and $S^{\prime }$).

Firstly, the local terrain slopes (always in the range dimension) must be computed. They can be easily derived since the elevation is known. Consider the SAR geometry shown in Figure 4 over a region with a given terrain slope α. Two adjacent pixels in the slant-range plane receive the backscattered signals from points *P* and $P^{\prime }$. The height difference Δh between both points can be directly obtained from the DEM by computing the local derivatives in the range direction, i.e.,

(9) $$\Delta h=h_{2}-h_{1}.$$

From trigonometry, we obtain the local slope between two adjacent pixels as [20],

(10) $$\alpha =\arctan\left(\frac{\sin\theta }{\frac{\Delta R}{\Delta h}+\cos\theta }\right),$$

where θ is the incidence angle and ΔR is the local slant range difference between the two pixels.

Due to the presence of varying topography, the filter must be very well adapted to the local variations in the slope. Therefore, it is necessary to segment the images into small patches that can be filtered properly.

**Figure 5:**
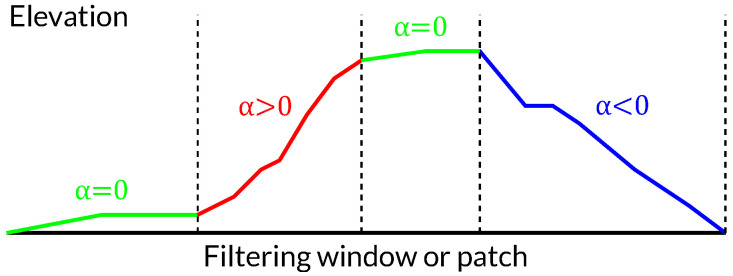
Example of filtering problem where a variety of slopes (α) is present in the same patch.

However, a problem arises if we have a topography like that shown in Figure 5. In this case, the selection of the slope, and hence the estimation of the spectral displacement, is not evident. Selecting the mean slope is not representative of the whole area, whereas selecting the maximum value could result in too coarse a filtering (which would entail an excessive loss of resolution), and selecting the minimum would not filter enough in some areas.

**Figure 6:**
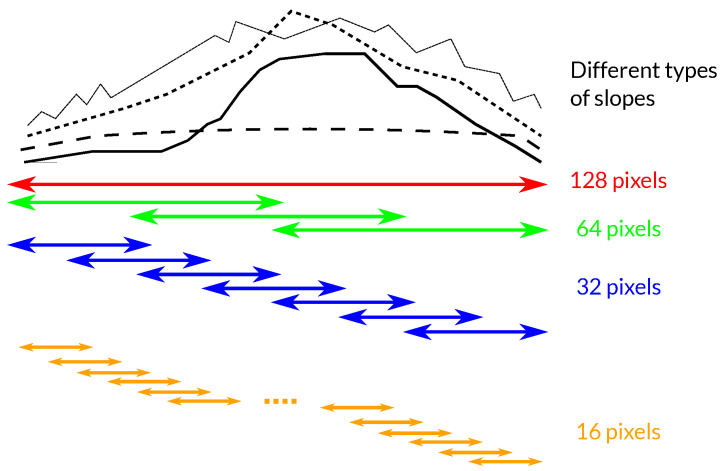
Representation of the proposed multi-scale range-filtering algorithm. An overlapping factor of 50% is represented.

In this regard, an optimal filtering can be obtained through a subdivision of the original window into smaller sub-blocks, resulting in a *multi-scale filtering*, so that the filter is always adapted to any kind of slope. If the topography exhibits strong spatial variations, smaller windows should provide better spectral shift estimations which, consequently, results in better filtering performance. On the contrary, large areas with uniform slopes benefit from larger blocks. Visually, this is represented in Figure 6. For simplicity, only four different window sizes are shown.

The proposed method is explained as follows. The algorithm works line by line by extracting a number of range pixels (i.e., a range interval). Each range interval is segmented into blocks of different sizes. Sizes of 128, 64, 32, and 16 pixels are proposed. Note that an overlap is introduced to avoid edge effects between adjacent filtered blocks, as represented in Figure 6. The spectral shift is obtained using geometrical parameters with Equation (4). The maximum displacement value is always selected and the range interval is filtered with all window sizes. Then, a quality criterion has to be established to determine which one performed best. The interferometric coherence is selected as the quality estimator since it provides a direct indication of the phase quality and is widely used in InSAR applications [21]. The expression of the coherence is

(11) γ=1N∑n=1NS1S2*1N∑n=1NS1S1*·1N∑n=1NS2S2*123(N>1),

where *N* is the number of averaged samples (or multi-look number) and *n* is the sample position/number. Usually, a square or rectangular window of a given size is used for its estimation. The magnitude of Equation (11), which ranges between 0 and 1, is usually employed as the estimator of the phase quality.

**Figure 7:**
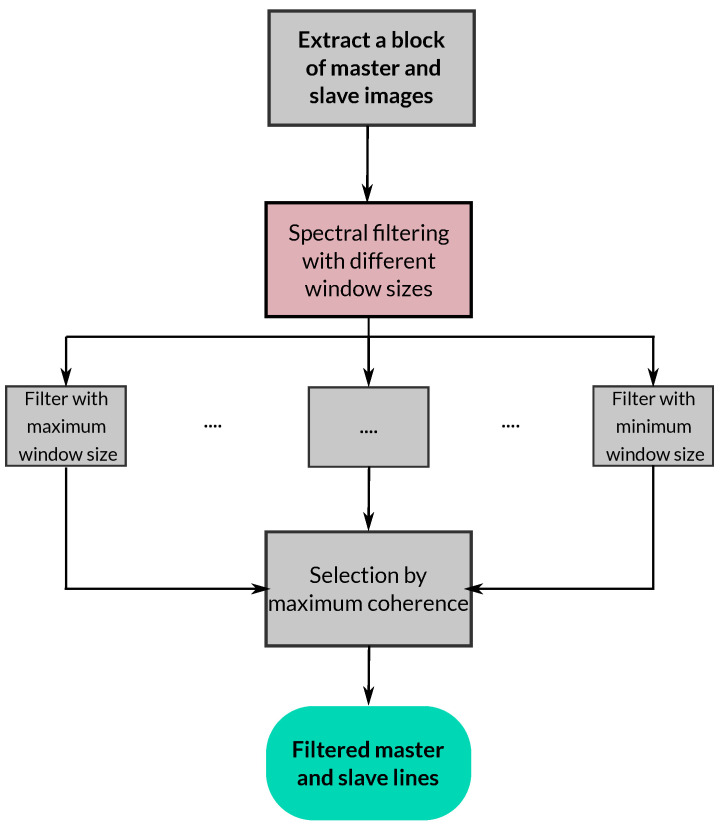
Block diagram of the proposed range-filtering method.

In this case, however, since the filtering is carried out only in the range direction, the quality estimator corresponds to the 1D coherence where only range pixels are used. It follows that the final values in the lines of the master and slave images are obtained as the ones for which the coherence is maximum. Note that to avoid the effect of the coherence estimation bias [21], which depends on the number of samples, coherence is always computed with a fixed window size. We also know that the effective number of averaged pixels (looks) may vary slightly once a line is filtered with the different window sizes. However, in practice, this difference is rather small so the 1D coherence provides a good quality estimator. In addition, it is worth mentioning that a maximization of the coherence in the range filter was also proposed in [22]. A general scheme of the filter is shown in Figure 7.

**Figure 8:**
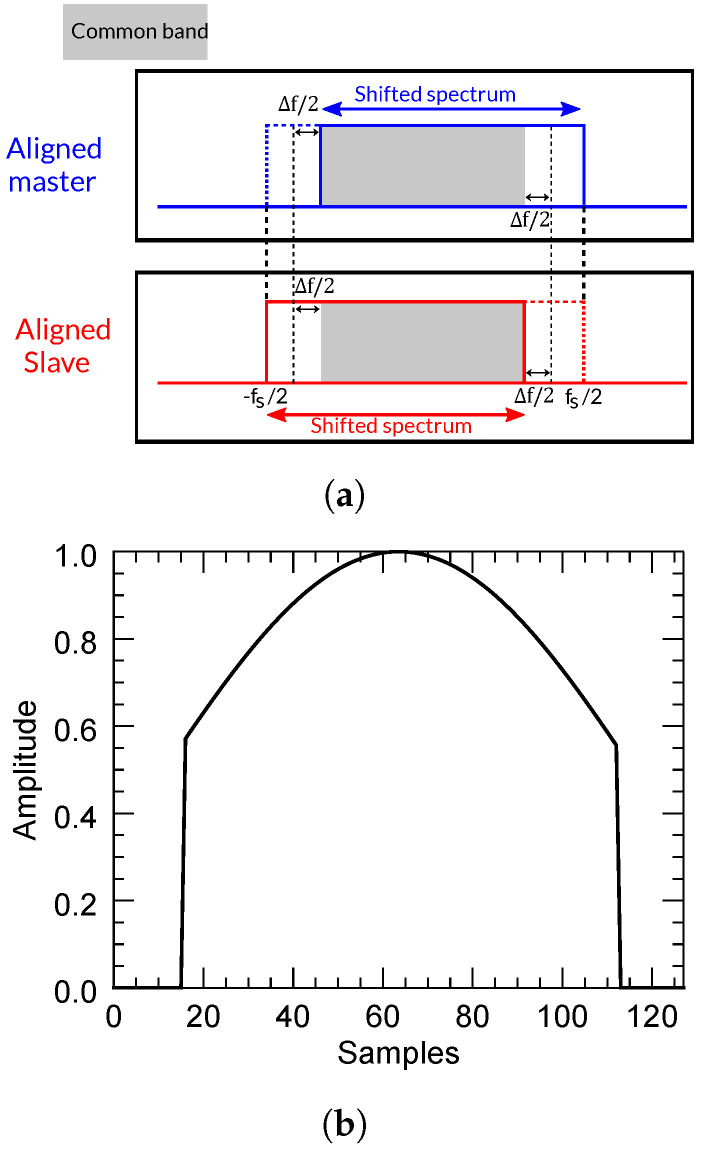
Low-pass spectral filtering after alignment of master and slave images. A spectral displacement of 30 samples is assumed. (**a**) Spectral alignment of master and slave images. (**b**) Spectral shape of the low-pass filter:a Kaiser window of 128 samples.

An important aspect of the proposed method is the construction of the low-pass or band-pass filter. Two different yet equivalent strategies are proposed. On the one hand, both the signals of the master and slave images can be demodulated using the topographic phase according to the process described in Section 2.3.2. In this case, a symmetric low-pass filter, which is identical for the master and the slave, is directly built according to Δf, i.e., we ’move’ the signals and we keep the same filter for both images. Note that the value of Δf in the frequency samples is easily obtained by

(12) $$\Delta f_{pixels}=N\cdot \frac{\Delta f}{f_{s}},$$

where $f_{s}$ is the sampling rate in the range dimension, which depends on the sensor, and *N* is the number of samples. This is illustrated in Figure 8. A Kaiser window with β=2.4 (RADARSAT-2 spectral weighting) is used. Because the spectrum of each image is shifted in opposite directions, the common band is aligned and the filter keeps the useful common band and removes the rest of each signal.

**Figure 9:**
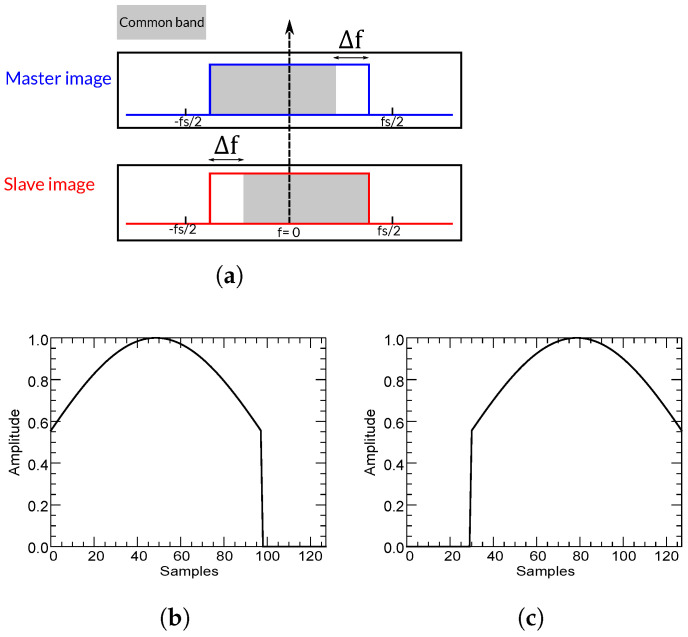
Band-pass spectral filtering of master and slave images. A spectral displacement of 30 samples is assumed. (**a**) Master and slave images’ spectra. (**b**) Spectral shape of the band-pass filter used for master image. (**c**) Spectral shape of theband-pass filter used for slave image.

On the other hand, if both signals are not demodulated, a similar yet inverse procedure is carried out, that is, we keep the signals and we adapt the filter to appropriately remove the non-common bands. In this case, a different and non-symmetric (reversed) filter is used for each signal. This is illustrated in Figure 9. The same Kaiser window (β=2.4 and 128 samples) and the same spectral shift of 30 samples are used.

Finally, for a better understanding of the proposed method, all the steps are summarized in Algorithm 1.
**Algorithm 1** Proposed Multi-scale Range-Filtering Method.Set maximum and minimum window sizes $W_{max}$ and $W_{min}$, e.g., $W_{max}=128$ and $W_{min}=16$ (pixels).For all image lines:
(a)Extract master and slave segments/sub-lines according to $W_{max}$ and remove original weighting window/filter. Note that this weighting window depends on the SAR sensor.
i.Compute all possible frequency shifts using Equation (4) and select the maximum value.ii.Convert the frequency shift to pixels using Equation (12).iii.Construct the filter/new weighting window according to the estimated shift (in pixels). See Figure 8 and Figure 9.iv.Filter master and slave segments (multiplication in frequency domain). If the low-pass version is chosen, images must be demodulated using Equation (7) before filtering and then modulated back using Equation (8).(b)Filter the same master and slave segments (still $W_{max}$ pixel size) with the remaining window sizes (e.g., 64, 32, and 16 pixels) following steps 2(a)-i:iv. This yields different $W_{max}$ pixel-filtered segments.(c)Compute the local 1D coherence using Equation (11) (using only range samples) of all the previously obtained filtered segments.Set as the final master and slave filtered segments the ones providing the maximum coherence.

## 3. Results

**Figure 10:**
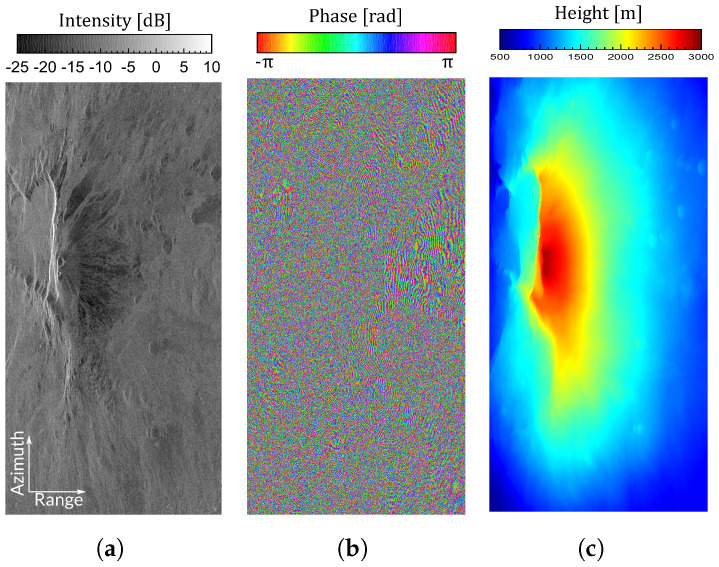
Master image intensity, original interferogram, and DEM height of the processed area (converted to slant-range coordinates). (**a**) Master intensity (dB). (**b**) Interferogram. (**c**) DEM height of the processed area.

To verify the performance of all methods including the proposed one, we analyzed the phase quality improvement achieved after range filtering a pair of images covering the area of Mount Etna (Sicily, Italy). Specifically, the data set was composed of two coregistered SLC (single-look complex) images acquired by RADARSAT-2 on 5 and 29 May 2008. They were acquired using Fine Quad Swath 8 (FQ8) mode, the near and far range incidence angles of which were 26.9${}^{\circ }$ and 28.7${}^{\circ }$, respectively. The processed image size was 2000 × 4000 pixels (range and azimuth, respectively) and the polarimetric channel was HH. The main system parameters used in the filtering process are detailed in Table 1. The intensity of the master image is represented in Figure 10a, the unfiltered interferogram (after subtracting the flat-Earth phase component) is shown in Figure 10b, and the DEM (SAR coordinates) providing the height data is depicted in Figure 10c.

**Figure 11:**
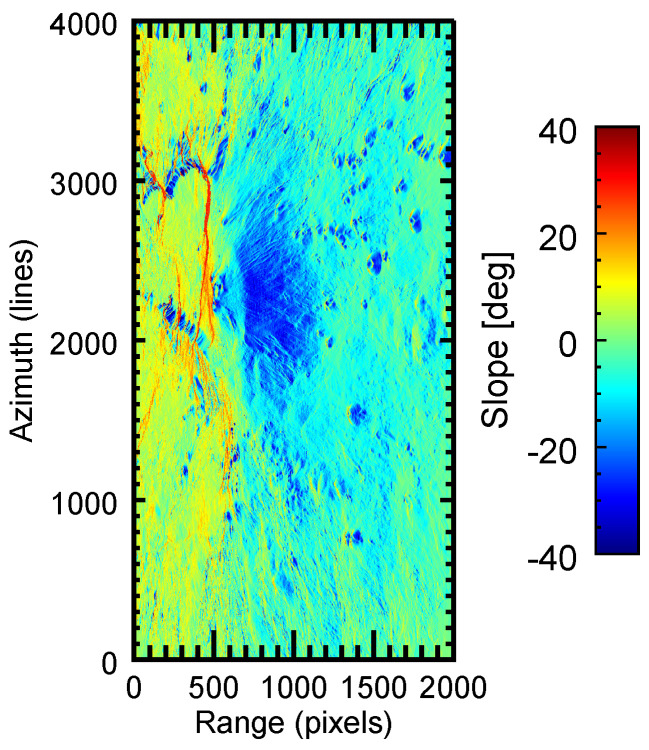
Local slopes of the terrain inslant-range direction of the test area.

Moreover, the slant-range slopes of the test scene are represented in Figure 11. It can be observed that this scene exhibited strong slopes throughout the whole area, especially around Mount Etna in the central part of the image.

It is important to mention that all the results discussed in this section are only related to the range-filtering process, that is, we only show the quality improvement after removing the baseline decorrelation. Concerning the adaptive method, blocks of 128 × 500 pixels (in range and azimuth dimensions, respectively) were progressively extracted and filtered, 35 azimuth lines in each block were averaged to compute the power spectrum, and both images were oversampled by a factor of 2 in the range dimension. Additionally, a minimum signal-to-noise ratio (SNR) threshold equal to 3, allowing the filter to proceed, was fixed. To show the impact of the window size (number of samples) on this method, its performance was also tested with a block size of 32 × 500 pixels, and 25 lines were averaged to compute the power spectrum. Concerning the method based on a constant flat DEM, each range line was divided into blocks of 128 pixels, and the spectral shift was estimated using Equation (4) with a fixed (null) slope. Images were also filtered with the conventional slope-adaptive method after including the demodulation in the topographic phase. In this case, a global spectral displacement Δf was selected (the one providing the maximum shift). Furthermore, the proposed slope-adaptive multi-scale algorithm was applied as a band-pass and a low-pass filter, following both strategies described in Section 2.3.3. Nevertheless, since they both provided the same results, we only show the results obtained with the low-pass version. Each extracted range line was filtered four times with windows of 128, 64, 32, and 16 pixels, and an overlap of 50% between adjacent blocks was set. To decide which block size performed better, the coherence along a line was estimated with a kernel of 15 samples.

**Figure 12:**
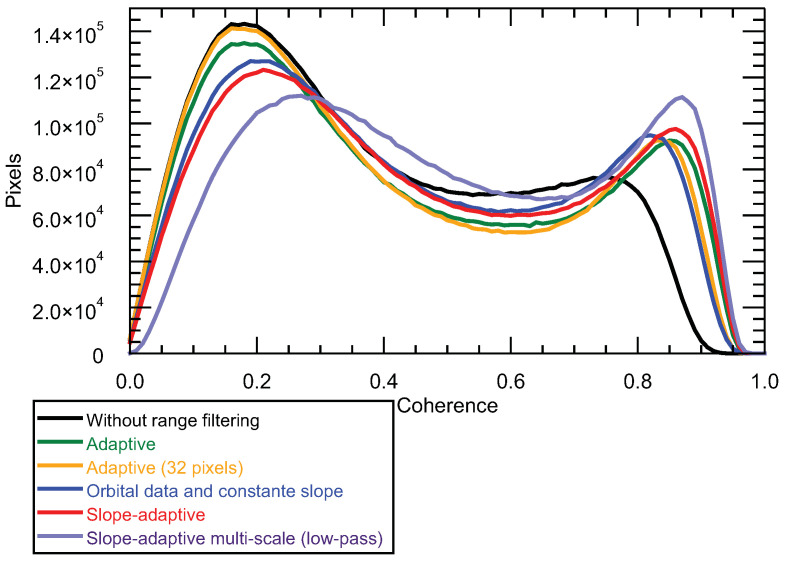
Coherence histograms after range filtering.

The overall quality improvement was assessed with the coherence histograms shown in Figure 12. A multi-look size of 9 × 5 pixels was used for coherence computation. It can be observed that all methods produced a clear improvement with respect to not filtering, as all histograms were displaced towards higher values. This also proves that the original data were significantly influenced by a geometrical decorrelation.

**Figure 13:**
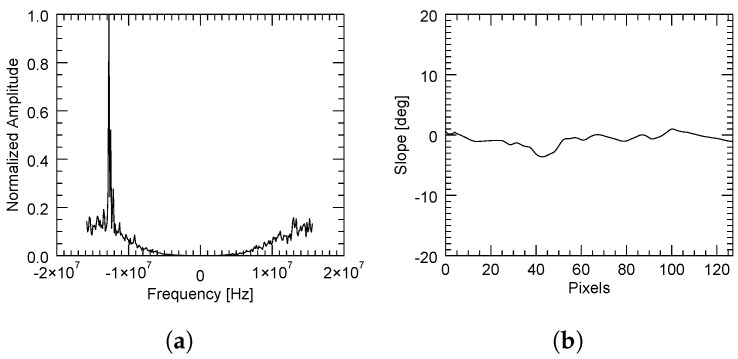
Computed normalized power spectrum (**a**) with the adaptive method in a region with an almost constant slope (**b**). A high SNR allows a clear identification of the peak (coherent area).

The adaptive method provided higher coherence values than the method based on a constant slope. This is because in high SNR areas, the computation of the power spectrum was accurate enough to yield a reliable estimate of the spectral displacement. However, it had the disadvantage of some lines not being filtered due to the reasons previously explained in Section 2.2. This can be illustrated by three different cases as follows. In the first case, when there was a sufficient correlation between the images, the spectral displacement was very well determined, as shown in Figure 13, where the slope was rather constant.

**Figure 14:**
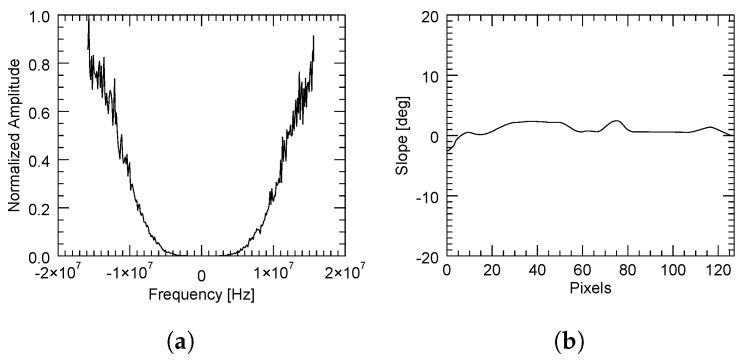
Computed normalized power spectrum (**a**) with the adaptive method in a region with an almost constant slope (**b**) but with strong decorrelation. A low SNR does not allow a clear identification of the peak.

**Figure 15:**
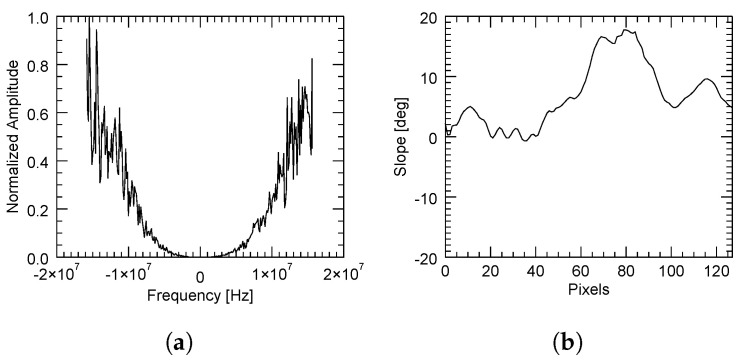
Computed normalized power spectrum (**a**) with the adaptive method in a region with a rapidly variant topography (**b**). A low SNR does not allow a clear identification of the peak.

Contrarily, a bad estimation of the spectral shift may come from either a flat but decorrelated area (for instance, due to the presence of vegetation) or a correlated zone with a strongly variant topography. These are, respectively, illustrated in Figure 14 and Figure 15, in which the average power spectrum is noisy and a dominant peak cannot be correctly identified.

**Figure 16:**
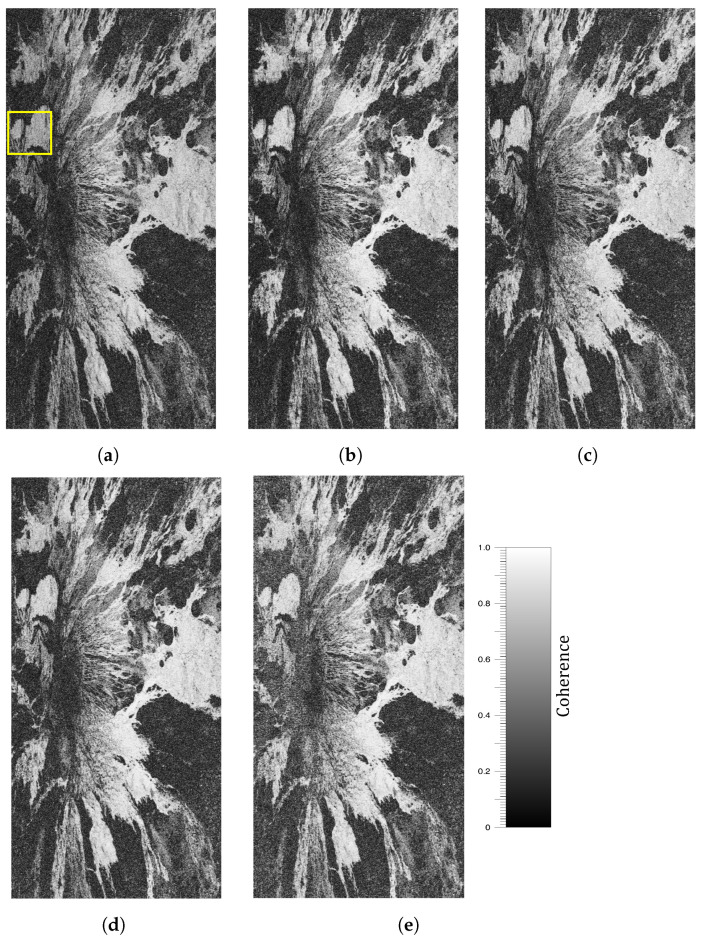
Coherence maps of the processed area after range filtering. (**a**) Original. (**b**) Adaptive method. (**c**) Method based on constant slope. (**d**) Slope-adaptive. (**e**) Multi-scale slope-adaptive. The yellow box denotes the specific region of interest analyzed in Figure 17 and Figure 18.

The improvement was also obvious in the resulting coherence maps of the processed area, which are depicted in Figure 16. All methods showed an overall increase in coherence throughout the whole area. However, a further gain was obtained when including the multi-scale filtering.

**Figure 17:**
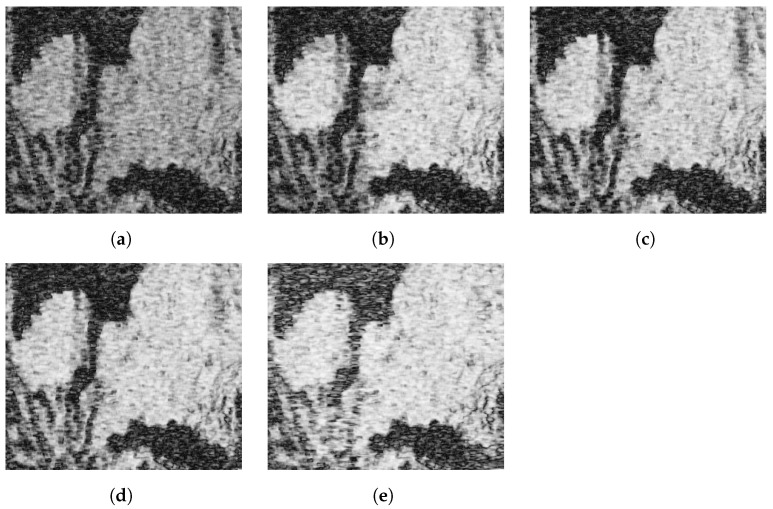
Coherence improvement after range filtering in a specific region of interest. (**a**) Original. (**b**) Adaptive method. (**c**) Method based on constant slope. (**d**) Slope-adaptive. (**e**) Multi-scale slope-adaptive.

**Figure 18:**
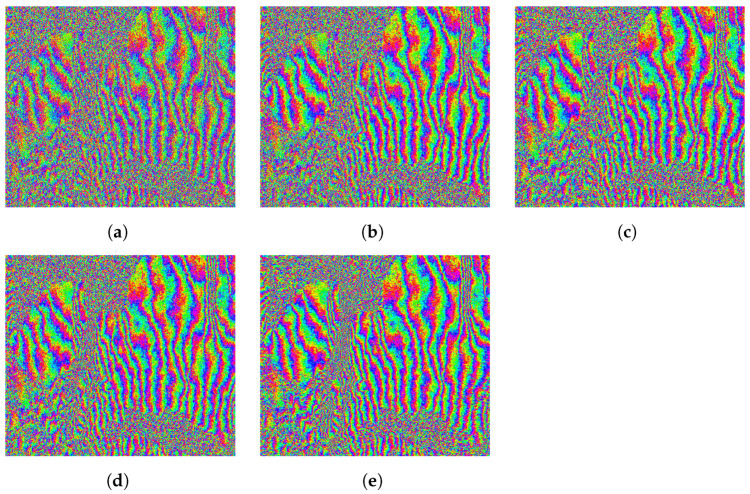
Phase quality improvement after range filtering in a specific region of interest. (**a**) Original. (**b**) Adaptive method. (**c**) Method based on constant slope. (**d**) Slope-adaptive. (**e**) Multi-scale slope-adaptive.

This is better visualized by looking at the area indicated by a yellow square in the original coherence map in Figure 16a. The coherences of this region of interest (RoI) are shown in Figure 17, whereas the corresponding interferometric phases are represented in Figure 18. It is clear that the fringes are sharper in the filtered data, so the global quality of the phase is improved, and hence the coherence is increased to a greater extent by the proposed method.

**Figure 19:**
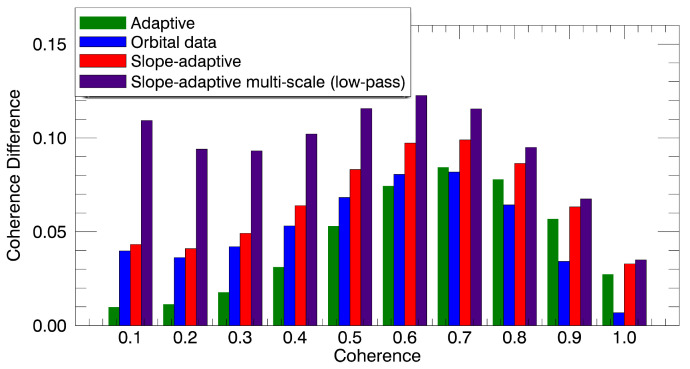
Coherence improvement for different intervals of coherence. Ten intervals were selected within [0,1].

As an additional comparison, the improvement provided by each method at different coherence intervals was evaluated. Specifically, 10 coherence intervals were selected between 0 and 1. As shown in Figure 19, the slope-adaptive methods provided the greatest improvement. Notably, the proposed algorithm was able to improve the coherence at all levels, outperforming the rest of the filters. The adaptive method exhibited the worst results in low-coherence areas, proving that this method is not able to filter areas strongly affected by other sources of decorrelation (improvement was almost negligible for coherence values below 0.3), for which the constant-slope orbit-based method provided some coherence improvements. On the contrary, the adaptive method performed better than the method based on orbits in highly coherent areas. In fact, it provided an improvement very similar to the slope-adaptive methods for coherence values greater than 0.8.

Quantitative measurement of the improvement after range filtering was provided by the so-called *phase residues* [23], which correspond to inconsistencies in the wrapped phase values and represent a way to identify erroneous measurements that could produce inaccuracies during the phase unwrapping step. Table 2 shows the remaining residues after range filtering with each method. The improvements in both the whole area and the specific RoI shown in Figure 18 are detailed.

Concerning the complete area, it was observed that the original number of residues was large, showing that the original phase was considerably degraded by noise. This is in line with the improvement offered by the adaptive method, which was the worst among all tested range filters as a result of the low-quality original interferogram (from which every spectral displacement was computed). The slope-adaptive method exhibited a greater improvement than the method based on orbits with a constant slope, proving that the inclusion of the slope information positively influences the filtering performance. The greatest improvement was clearly obtained with the proposed multi-scale strategy. By looking at Table 2, it can be seen that the number of remaining residues was greatly reduced. In fact, the improvement in terms of residues was close to double that of the slope-adaptive method, showing that the multi-scale filter completely adapts to the local topography, so the filtering performance is greatly enhanced. A major improvement of the proposed method was also in the specific RoI obtained. As shown in Table 2, among all the filters, the proposed slope-adaptive multi-scale method offered the best results since it was able to reduce the number of remaining phase residues to a greater extent, proving that the proposed methodology maximizes the range-filtering performance.

**Figure 20:**
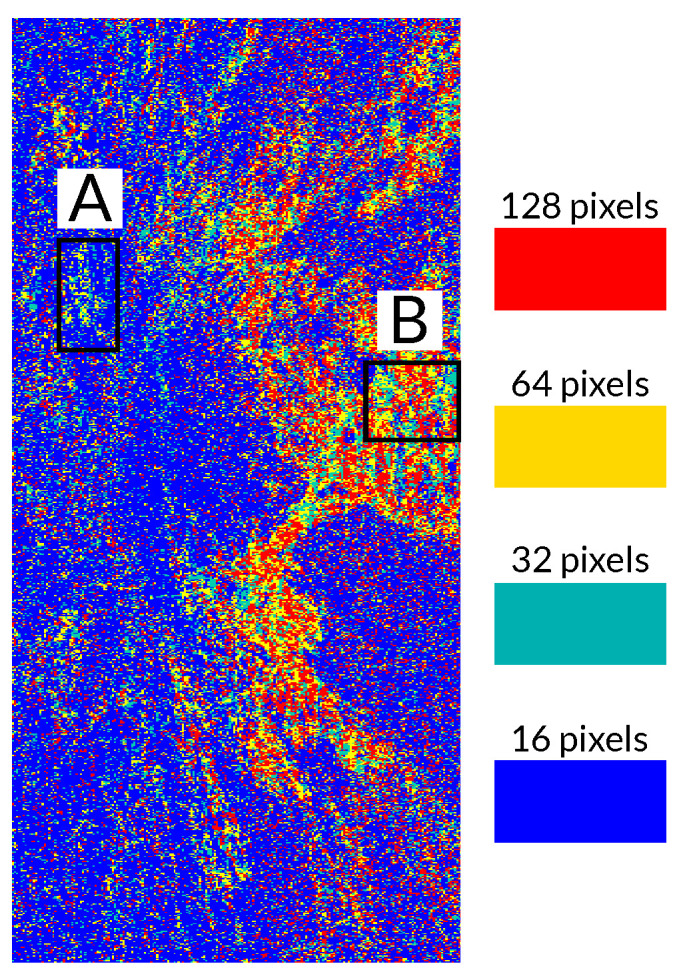
Map of the block sizes providing the best filtering results in the processed area. Region A is located near the summit area of Mount Etna. Region B is a flat area.

Finally, it is interesting to visualize the window size that provided the best results (i.e., the best coherence) in the processed area, so that the utility of testing multiple window sizes is justified. The color map in Figure 20 shows the window size that provided the best filtering results in the whole processed area. By comparing Figure 11 and Figure 20 it can be deduced that there is a direct relationship between the filtering window size and the local terrain slope, as highlighted in Section 2. In fact, small windows (especially of 16 pixels) produced the best results in most parts of the scene. This was expected since the images corresponded to a mountainous area where strong terrain slopes are present. Only flatter areas benefited from larger window sizes (128 and 64 pixels).

**Figure 21:**
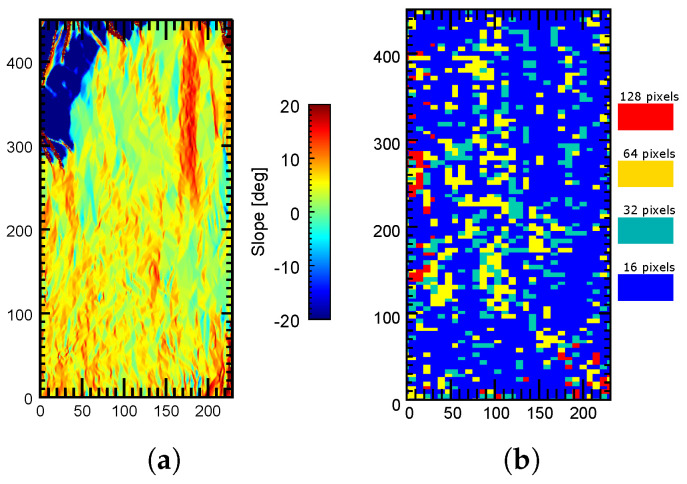
Filtering window sizes providing the best results in an area where strong terrain slopes are present. (**a**) Local terrain slopes in the region of interest. (**b**) Window sizes providing the best filtering results in the region of interest A.

**Figure 22:**
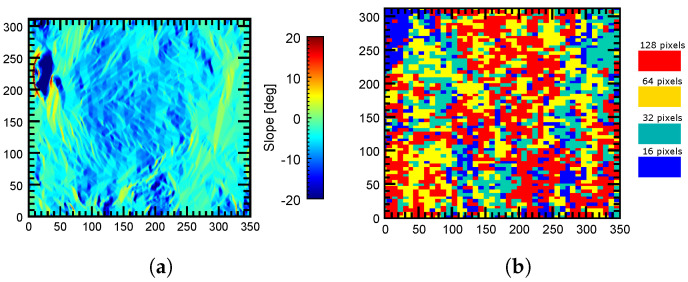
Filtering window sizes providing the best results in a flat area. (**a**) Local terrain slopes in the region of interest. (**b**) Window sizes providing the best filtering results in the region of interest B.

This is better illustrated when we compare the filtering window sizes and the slopes of the two regions, labeled A and B in Figure 20. The first one (A) is located near the summit area of Mount Etna, so strong terrain slopes are present, as shown in Figure 21a. In this case, by looking at Figure 21b, the best filtering results were obtained with small windows (16 and 32 pixels have a clear dominance in this area). In fact, the largest window (128 pixels) was rarely used. On the contrary, in the flat area (B) shown in Figure 22a, larger window sizes (128 and 64 pixels) seemed to perform better since more image blocks were filtered with these window sizes, as shown in Figure 22b.

## 4. Discussion

A revision of different, state-of-the-art range-filtering methods, has been carried out in this work. We have shown the difficulties that these methods face, especially in areas strongly influenced by topography.

The conventional and widely used adaptive method has an important double drawback, that is, besides the difficulty of accurately estimating the spectral shift in areas with topography, the method is highly dependent on the original quality of the interferometric phase since it cannot estimate the shift if other sources of decorrelation are present. In other words, the filtering may be either inaccurate or unfeasible. However, it has the advantage of not requiring any external data. Concerning the method based on a constant DEM (i.e., a constant terrain slope) and satellite orbits, it is clearly limited if strong topographic variations are present because they are not taken into account. As a consequence, an inaccurate estimation of the spectral shift is computed and its solution is not optimum. The main advantage is that the algorithm is considerably faster than other methods and it should provide good results in flat areas.

It has been shown that in areas influenced by topography, slope-adaptive methods are undoubtedly needed. In this regard, the filter proposed in [17], assuming that an external DEM is provided, offers good overall results. However, it only partially exploits the slope information derived from the DEM. In this regard, the proposed range-filtering method has shown that it can overcome all the limitations of the other filters and is able to extensively suppress the geometrical decorrelation of the interferometric pair. We have shown that the size of the filtering window (i.e., the number of samples used in the filtering process) has an influence on the final results, so the proposed multi-scale strategy automatically adapts the filter to all types of surface variations. Consequently, better performance is always obtained regardless of the smoothness of the topography, and even some useful interferometric fringes may be properly recovered in areas where the other filters are unable to achieve this. The main drawback is that the proposed method is computationally slower than the other ones, but it optimizes the range-filtering step in complicated areas with strong topographic variations.

In summary, we have seen that the removal of the geometrical decorrelation improves the phase quality and globally increases the coherence between the interferometric image pair. However, it is important to point out that besides improving the quality of the InSAR phase and the other products derived from it, such as the topography of an area or surface displacements in the case of differential interferometry, obtaining accurate coherence measurements is crucial in other applications. In fact, there is a wide variety of scientific applications that directly use coherence as an input feature and assume that all decorrelation factors that depend on the sensor parameters and acquisitions’ geometry are completely suppressed [24]. A geometrical decorrelation is a type of sensor- and geometry-dependent term, as we have studied in this work. However, if properly compensated, the resulting coherence will not only be increased but, more importantly, also better estimated and only related to the specific characteristics of the imaged scene so it can be properly exploited. Coherence-based applications include physical parameter estimations (such as vegetation height, biomass, etc. [5,6,7,8]) and land-cover classifications [25,26], among others. The removal of a geometrical decorrelation using an accurate range-filtering method will therefore be beneficial not only for interferometry but also for other practical applications that require coherence data that are not affected by decorrelation sources that are not dependent on the scene variables to be estimated.

## Figures and Tables

**Table 1:** Processed interferometric pair characteristics.

Master date	5 May 2008
Slave date	29 May 2008
Perpendicular baseline [m]	586.547
Range bandwidth [MHz]	30.02442 MHz
DEM resolution [m]	10
Range spectral weighting	Kaiser-Bessel window with β = 2.4

**Table 2:** Performance analysis of the different range-filtering methods in the full processed area and the specific RoI shown in Figure 18.

	Residue Number	Improvement
**Full Area**		
Original	1,512,593	–
Adaptive method	1,370,349	9.4%
Method based on constant slope	1,317,685	12.89%
Slope-adaptive	1,273,586	15.80%
Multi-scale slope-adaptive	1,085,287	28.24%
**Specific RoI**		
Original	28,960	–
Adaptive method	21,524	25.67%
Method based on constant slope	20,558	29.01%
Slope-adaptive	195,46	32.51%
Multi-scale slope-adaptive	16,998	41.30%

## References

[1] Hanssen R. (2001). Radar Interferometry: Data Interpretation and Error Analysis.

[2] Zebker H., Villasenor J. (1992). Decorrelation in interferometric radar echoes. IEEE Trans. Geosci. Remote Sens..

[3] Bamler R., Hartl P. (1998). Synthetic Aperture Radar Interferometry. Inverse Probl..

[4] Gatelli F., Monti-Guarnieri A., Parizzi F., Pasquali P., Prati C., Rocca F. (1994). The Wavenumber Shift in SAR Interferometry. IEEE Trans. Geosci. Remote Sens..

[5] Kugler F., Schulze D., Hajnsek I., Pretzsch H., Papathanassiou K.P. (2014). TanDEM-X Pol-InSAR performance for forest height estimation. IEEE Trans. Geosci. Remote Sens..

[6] Olesk A., Praks J., Antropov O., Zalite K., Arumae T., Voormansik K. (2016). Interferometric SAR Coherence Models for Characterization of Hemiboreal Forests Using TanDEM-X Data. Remote Sens..

[7] Chen H., Cloude S.R., Goodenough D.G. (2016). Forest Canopy Height Estimation Using Tandem-X Coherence Data. IEEE J. Sel. Top. Appl. Earth Obs. Remote Sens..

[8] Lopez-Sanchez J.M., Vicente-Guijalba F., Erten E., Campos-Taberner M., Garcia-Haro F.J. (2017). Retrieval of vegetation height in rice fields using polarimetric SAR interferometry with TanDEM-X data. Remote Sens. Environ..

[9] Sansosti E., Berardino P., Manunta M., Serafino F., Fornaro G. (2006). Geometrical SAR image registration. IEEE Trans. Geosci. Remote. Sens..

[10] Serafino F. (2006). SAR image coregistration based on isolated point scatterers. IEEE Geosci. Remote Sens. Lett..

[11] Prati C., Rocca F. (1993). Improving slant-range resolution with multiple SAR surveys. IEEE Trans. Aerosp. Electron. Syst..

[12] Smith B. (2000). Generalization of spatially variant apodization to noninteger Nyquist sampling rates. IEEE Trans. Image Process..

[13] Castillo-Rubio C., Llorente-Romano S., Burgos-Garcia M. (2007). Robust SVA method for every sampling rate condition. IEEE Trans. Aerosp. Electron. Syst..

[14] Iglesias R., Mallorqui J.J. (2013). Side-Lobe Cancelation in DInSAR Pixel Selection with SVA. IEEE Geosci. Remote Sens. Lett..

[15] Kampes B.M. (1999). Delft Object-Oriented Radar Interferometric Software: Users Manual and Technical Documentation.

[16] Kampes B.M., Hanssen R.F., Perski Z. Radar Interferometry with Public Domain Tools. Proceedings of the FRINGE’03: Advances in SAR Interferometry from ERS and ENVISAT Missions.

[17] Davidson G., Bamler R. (1999). Multiresolution phase unwrapping for SAR interferometry. IEEE Trans. Geosci. Remote Sens..

[18] Bamler R., Davidson G. (2000). Method of Correcting an Object-Dependent Spectral Shift in Radar Interferograms. US Patent.

[19] Farr T.G., Rosen P.A., Caro E., Crippen R., Duren R., Hensley S., Kobrick M., Paller M., Rodriguez E., Roth L. (2007). The Shuttle Radar Topography Mission. Rev. Geophys..

[20] Wang T., Liao M., Perissin D. (2010). InSAR Coherence-Decomposition Analysis. IEEE Geosci. Remote Sens. Lett..

[21] Touzi R., Lopes A., Bruniquel J., Vachon P. (1999). Coherence estimation for SAR imagery. IEEE Trans. Geosci. Remote Sens..

[22] Colesanti C., De Zan F., Ferretti A., Prati C., Rocca F. Generation of DEM with sub-metric vertical accuracy from 30’ ERS-Ensvisat pairs. Proceedings of the ESA FRINGE Workshop on ERS SAR ASAR Interferometry.

[23] Goldstein R.M., A H., Werner C.L. (1988). Satellite Radar Interferometry: Two-Dimensional Phase Unwrapping. Radio Sci..

[24] Rizzoli P., Dell’Amore L., Bueso-Bello J., Gollin N., Carcereri D., Martone M. (2022). On the Derivation of Volume Decorrelation from TanDEM-X Bistatic Coherence. IEEE J. Sel. Top. Appl. Earth Obs. Remote Sens..

[25] Jacob A.W., Vicente-Guijalba F., Lopez-Martinez C., Lopez-Sanchez J.M., Litzinger M., Kristen H., Mestre-Quereda A., Ziółkowski D., Lavalle M., Notarnicola C. (2020). Sentinel-1 InSAR Coherence for Land Cover Mapping: A Comparison of Multiple Feature-Based Classifiers. IEEE J. Sel. Top. Appl. Earth Obs. Remote Sens..

[26] Mestre-Quereda A., Lopez-Sanchez J.M., Vicente-Guijalba F., Jacob A.W., Engdahl M.E. (2020). Time-Series of Sentinel-1 Interferometric Coherence and Backscatter for Crop-Type Mapping. IEEE J. Sel. Top. Appl. Earth Obs. Remote Sens..

